# Tissue-specific variations of piperine in ten populations of *Piper longum* L.: bioactivities and toxicological profile

**DOI:** 10.1038/s41598-024-52297-9

**Published:** 2024-03-01

**Authors:** Protha Biswas, Devendra Kumar Pandey, Mahipal S. Shekhawat, Abhijit Dey, Tabarak Malik

**Affiliations:** 1 Department of Life Sciences, Presidency University, Kolkata, India; 2 Department of Biotechnology, Lovely Faculty of Technology and Sciences, Lovely Professional University, Phagwara, India; 3 Plant Biotechnology Unit, Kanchi Mamunivar Government Institute for Postgraduate Studies and Research, Puducherry, India; 4 Department of Biomedical Sciences, Institute of Health, Jimma University, Jimma, Ethiopia

**Keywords:** Biochemistry, Biotechnology, Plant sciences

## Abstract

*P. longum* L., one of the most significant species of the genus Piperaceae, is most frequently employed in Indian-Ayurvedic and other traditional medicinal-systems for treating a variety of illnesses. The alkaloid piperine, is the key phytoconstituent of the plant, primarily responsible for its’ pharmacological-impacts. The aim of the study is to analyse the intra-specific variation in piperine content among different chemotypes (PL1 to PL 30) and identify high piperine yielding chemotype (elite-chemotype) collected from 10 different geographical regions of West Bengal by validated HPTLC chromatography method. The study also focused on the pharmacological-screening to better understand the antioxidant activity of the methanol extracts of *P. longum* by DPPH and ABTS radical-scavenging activity and genotoxic activity by *Allium cepa* root tip assay. It was found that the *P. longum* fruit chemotypes contain high amount piperine (highest 16.362 mg/g in chemotype PL9) than the stem and leaf chemotypes. Both DPPH and ABTS antioxidant assays revealed that *P. longum* showed moderate radical-scavenging activity and the highest activity was found in PL9 (fruit) chemotype with IC_50_ values of 124.2 $\pm 0.97$ and 104 $\pm 0.78$ µg/ml respectively. The *A. cepa* root tip assay showed no such significant genotoxic-effect and change in mitotic-index. The quick, reproducible, and validated HPTLC approach offers a useful tool for determining quantitative variations of piperine among *P. longum* chemotypes from different geographical-regions and also according to the different tissues and choose elite genotypes with high piperine production for continued propagation and commercialization for the pharmaceutical sector. Additionally, the plant's *in-vitro* antioxidant property and lack of genotoxicity directly supports its’ widespread and long history of use as a medicinal and culinary plant.

## Introduction

**Figure 1:**
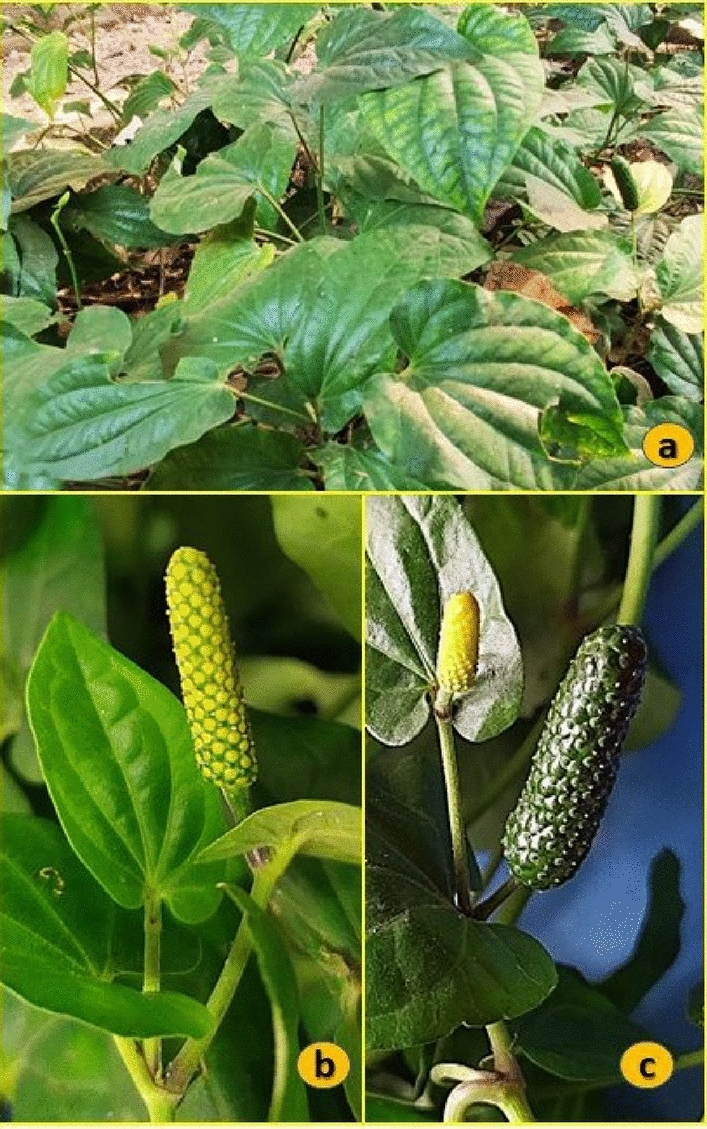
*P. longum* plant: (**a**) habit of the plant (**b**) immature fruit, (**c**) mature fruit.

*Piper longum*, often known as "long-pepper" or "Pippali," is a perennial shrub or herbaceous vine that belongs to the family Piperaceae (Fig. 1). It is indigenous to the Indo-Malaya region and is widely distributed throughout the tropical and subtropical regions of the world, including the Indian subcontinent, Sri Lanka, the Middle East, and America^1^. The fruits are mostly used in food as spices and preservatives, but they are also an effective treatment for bronchitis, cough, colds, snakebites, scorpion stings, and other ailments in a variety of traditional medical systems. They are also used as a form of contraception^1^. The Ayurvedic medical system mentions the use of *P. longum*, and the literature that is currently available suggests using the unripe fruit of *P. longum* for a variety of ailments, including respiratory problems, digestive problems, disorders of metabolic imbalance, aphrodisiacs, emmenagogues, circulatory stimulants, and analgesics^2^. *P. longum* is a popular herb among researchers because of its long history of usage, effectiveness in treating a variety of illnesses, and presence of certain alkaloids, which support the findings of science.

**Figure 2:**
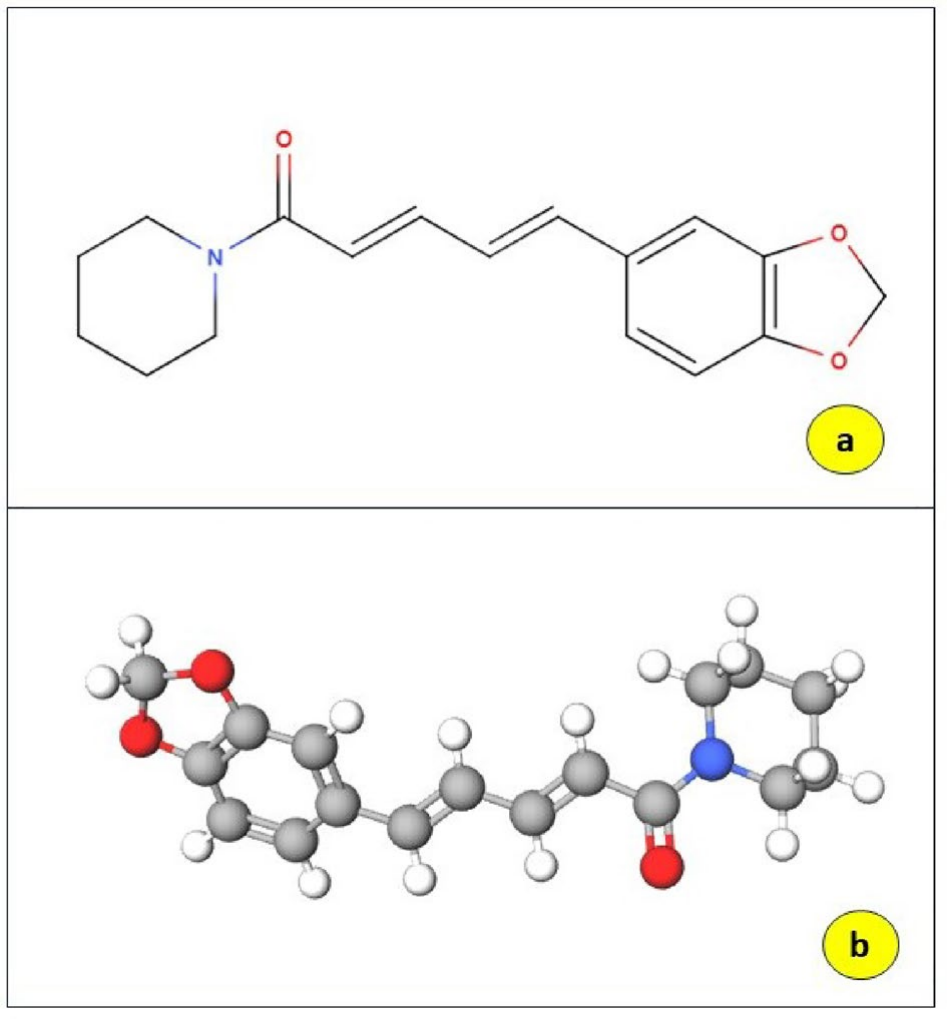
Chemical structure of piperine (**a**) 2D structure, (**b**) 3d structure (Downloaded from https://pubchem.ncbi.nlm.nih.gov).

The alkaloid piperine (Fig. 2), which gives *P. longum* its pungency, is known to be present in this herb. *P. longum*, which is well-recognized for being a significant bio-enhancer, is the well-known component in the herbal formulation known as "Trikatu"^3^. Due to its many properties, including those as an antioxidant, anticancer, antihypertensive, analgesic, anti-asthmatics, antipyretic, anti-inflammatory, anti-diarrheal, anxiolytic, hepato-protective, antispasmodic, antidepressant, immunomodulatory, antibacterial, antifungal, antithyroid, antiapoptotic, antimutagenic, anti-spermatogenic and antimetastatic, this substance has caught the interest of medicinal researchers and health specialists^4^. The most prevalent phytochemical, piperine, was first discovered by Hans Christian Ørsted in 1819. He found a yellow, crystalline substance with the chemical formula C_17_H_19_NO_3_ with a melting point between 128 and 130 °C. The chemical's structure was subsequently clarified, and its IUPAC designation is (2E, 4E) 5-(benzo[d] [1,3] dioxol-5-yl] 1-(piperidin-1-yl) penta-2,4-dien-1-one. During hydrolysis (acidic/basic), piperine, which is naturally weakly basic, can be transformed into piperic acid and piperidin^5^. Between the 5-(3,4-methylenedioxyphenyl) moiety and piperidine, a conjugated aliphatic chain serves as a connecting structure). As a result, piperine stands out as a special and superior molecule that has the best characteristics for its propensity to attach to the CYP-450 enzyme, an essential hemeprotein that aids in the metabolism of xenobiotics and other drugs^6^.

Attempts to isolate piperine compounds are currently being widely receiving a lot of attention of the researchers. Unfortunately, approaches to obtain pure chemicals necessitate time-consuming, ineffective procedures, and have little economic benefit, especially when manufactured on a big scale. For the identification of bioactive chemicals, numerous organisations, including ASTA, AOAC, and ISO, have suggested sensitive and accurate analytical procedures, such as TLC (Thin Layer Chromatography), HPLC (High Performance Liquid Chromatography), and spectrophotometric methods, among others. Advancements in planar chromatography, such as high-end imaging, photo-documentation, and densitometry in HPTLC (High Performance Thin Layer Chromatography) system, have recently been implemented under such analytical studies to ensure the reproducibility^7^. There are very few literatures found on quantification of piperine in *P. longum*. In the previous investigations, HPTLC was used to quantify piperine in roots^8^, fruits^9^ and Ayurvedic formulations^10^ of *P. longum*. The samples for these investigations, however, were only taken from a restricted number of neighbouring places. Because of this, it is impossible to evaluate the current natural fluctuations in the content of secondary metabolites and bioactivity in *P. longum* natural populations due to restrictions on the collecting zones and sample size.

Oxidative stress has a role in many illnesses such as (neurodegenerative and cardiovascular diseases, diabetes, cancer, digestive problems and metabolic syndrome) as a cause or a consequence^11,12^. Natural antioxidants derived from botanicals are essential for treating disorders brought on by oxidative stress^13^. According to studies, *P. longum* water extracts and its amide alkaloids had strong antioxidant properties, and a notable scavenging impact on superoxide, 1,1-diphenyl-2-picrylhydrazyl, and 2,2-diphenyl-1, (2,4,6-trinitrophenyl) hydrazyl (DPPH) radicals^14,15^.

Despite being used for decades, traditional herbal remedies' toxicity is frequently not fully understood, and some therapeutic plants can be quite dangerous to people's health. To examine chromosomal abnormalities brought on by various chemical poisons, the *Allium cepa* test is a popular and very sensitive assay^16^.

This study describes the variation of bioactive standard compound piperine in 30 different chemotypes of *P. longum* plant based on its geographical as well a tissue specific variation. The antioxidant and genotoxic properties of the plant also studied to prove the claim as ethnomedicinal plant and future use in therapeutics.

## Results

### HPTLC quantification

**Figure 3:**
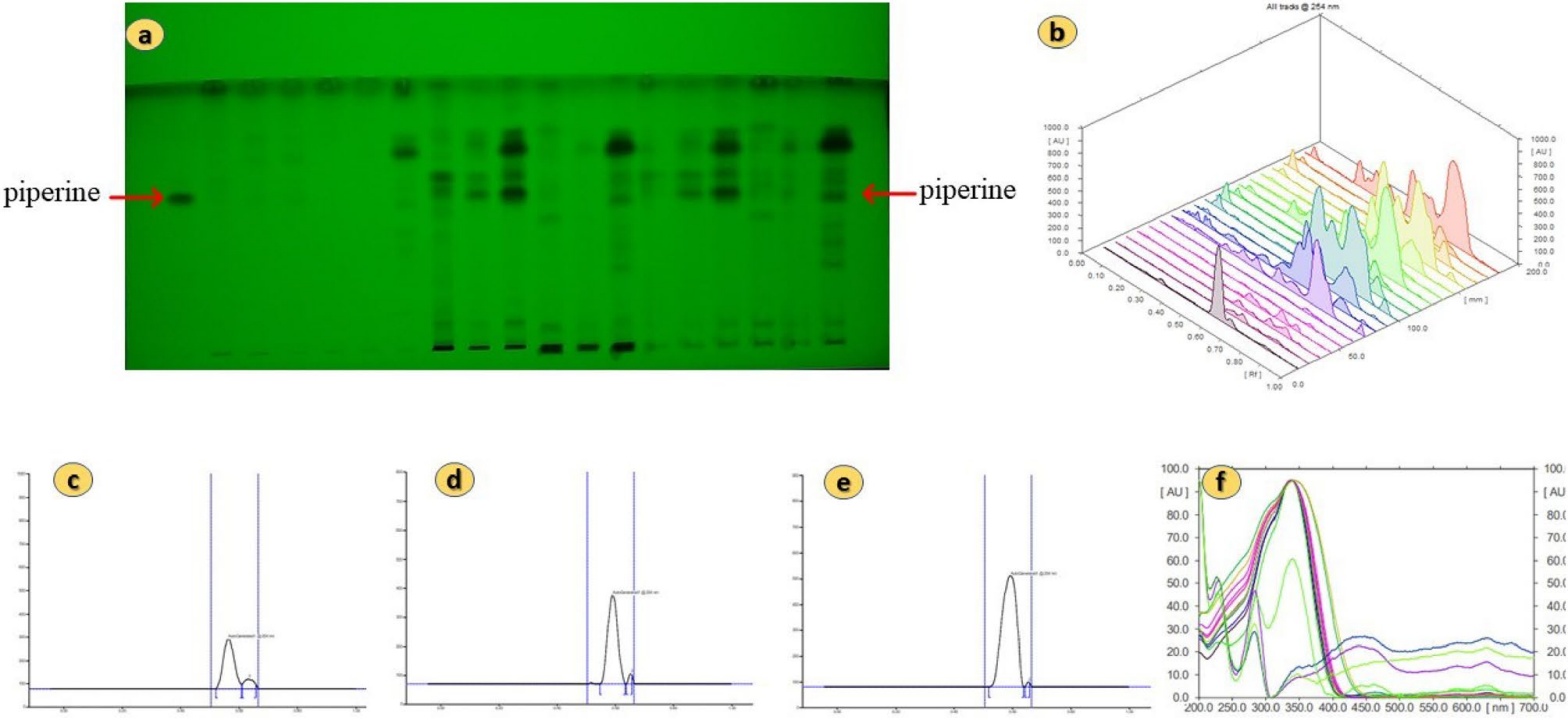
(**a**) Representative HPTLC fingerprint of piperine from first 18 chemotypes with Rf 0.63 resolved at 254 nm, (**b**) 3D densitogram of piperine and P*. longum* extracts, (**c**–**e**) chromatogram of *P. longum* leaf, stem and fruit (chemotype PL7, PL8, PL9), [also provided in Fig. S3 as PL7, PL8 and PL9] (**f**) overlay spectra.

**Figure 4:**
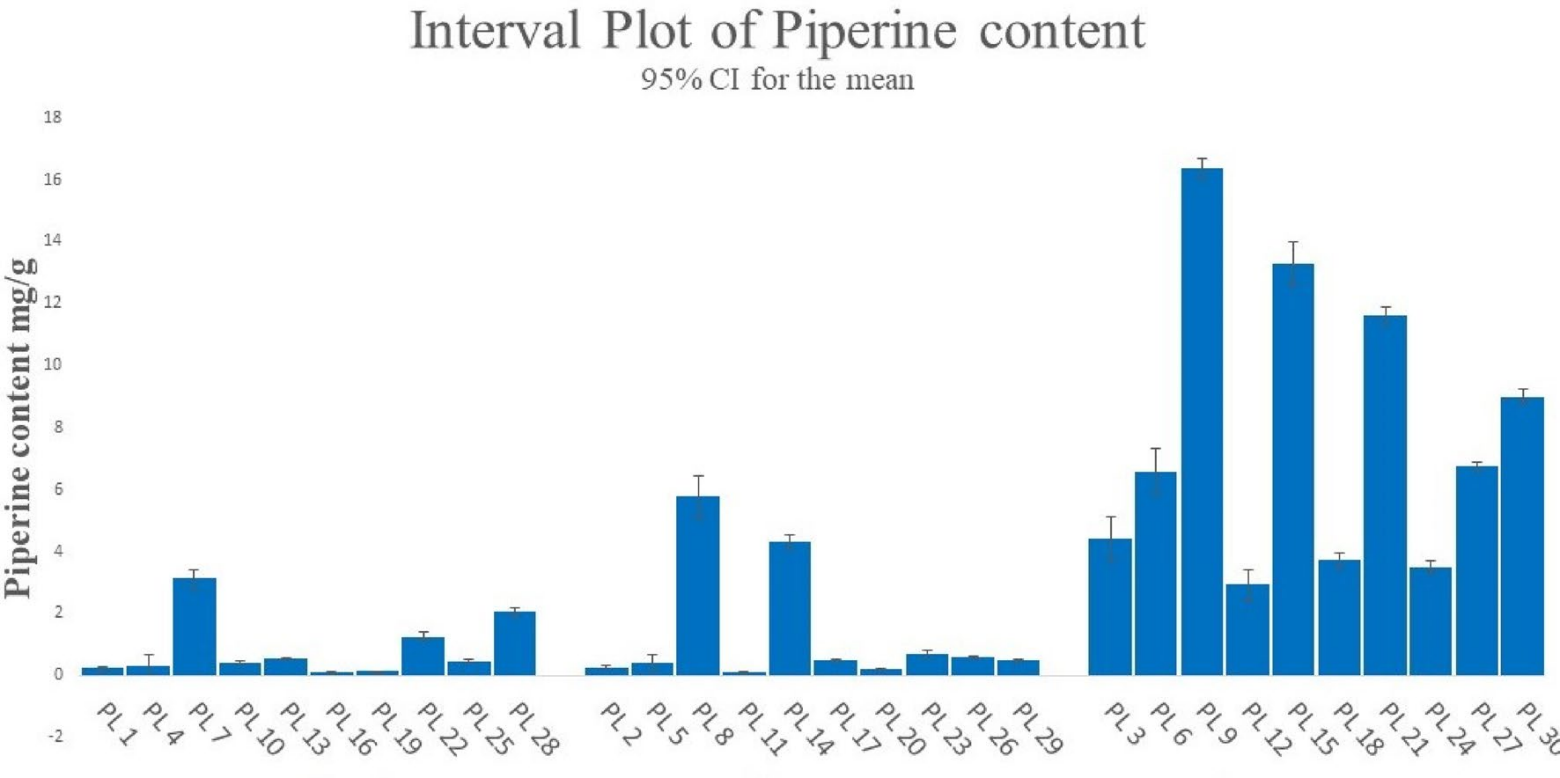
Tissue specific variation of piperine contents quantified by HPTLC in collected chemotypes of *P. longum.*

**Figure 5:**
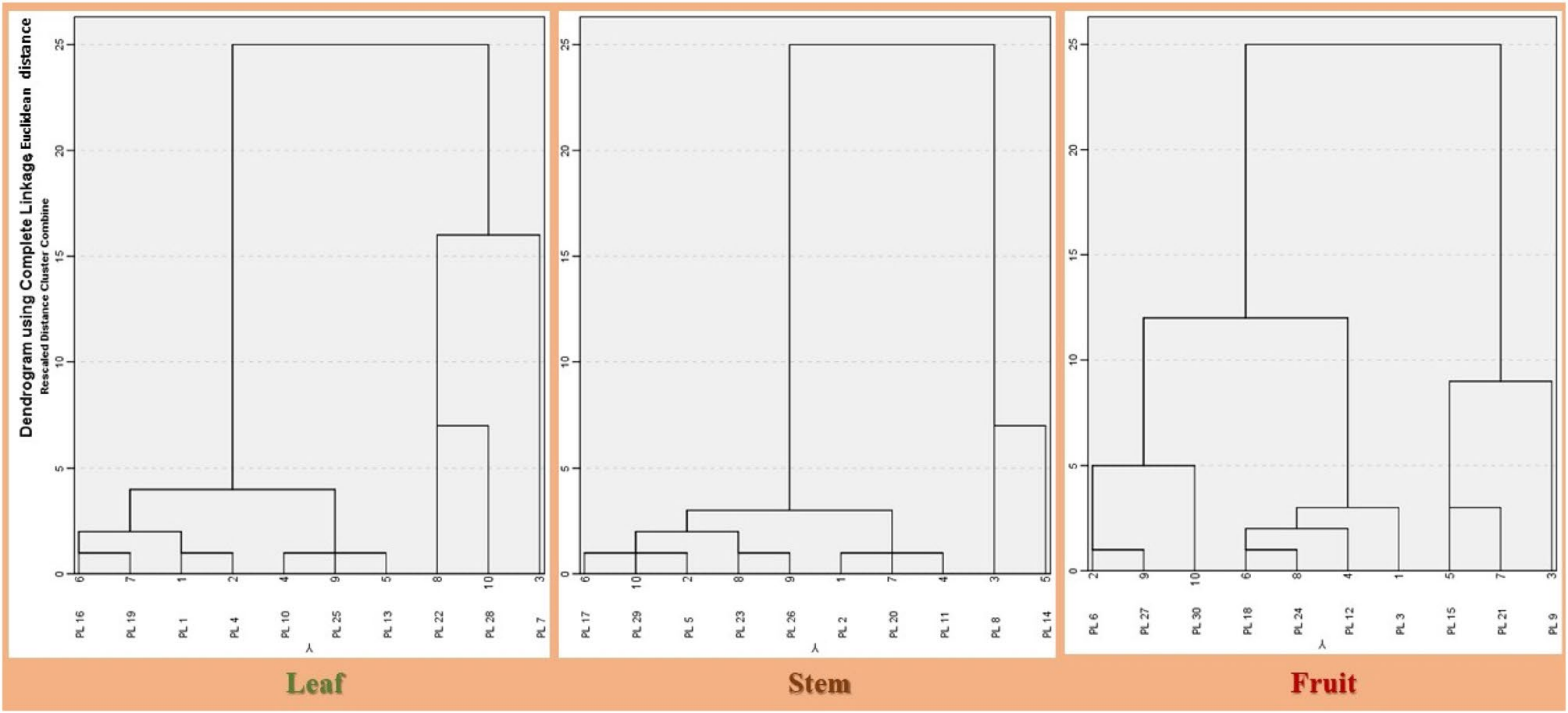
Tissue specific dendogram based on piperine content in collected chemotypes revealed four major clusters in each tissue. Cluster I represent elite chemotype.

The piperine component was successfully separated in the current experiment using the developing solvent toluene: ethylacetate: diethyl ether (6:3:1, v/v). The 3-D chromatograms and HPTLC fingerprints of the standard piperine compound were shown in Fig. S1. The separated band of the standard compound with the plant samples was verified with the assistance of the matching Rf values in their scanning densitometric chromatograms. The HPTLC-densitogram of a reference chemical, resolution of piperine in *P. longum* elite samples, and overlay spectra of the piperine compound with plant samples are displayed in Fig. 3. All of the plant samples' densitogram patterns—obtained from both the reference chemical and all of the plant samples (Fig. S3)—showed that the peak associated with Rf 0.63 could be superimposed in each sample of a plant. The quantity of piperine in *P. longum* chemotype extracts was investigated using the current methodology (Table 1). The *P. longum* chemotypes PL9 (collected from South 24 Pgs, West Bengal) and PL21 and PL15 (collected from Bardhaman, and Nadia) were found to have the highest amounts of piperine, respectively. All the three chemotypes are fruit samples. Piperine was present in the least amount in chemotype PL 16.30 chemotypes of *P. longum* had piperine content ranging from 0.109 to 13.362 mg/g. From all the collected *P. longum* chemotypes the fruit samples found to contains high amount of piperine, then the stems and leaves respectively from all the populations. Figure 4 compares the amounts of piperine in 30 different *P. longum* chemotypes as measured by HPTLC. The dendrogram based on piperine content in *P. longum* chemotypes is shown in Fig. 5.

**Table 1:** Piperine content in different chemotypes of *P. longum* collected from different geographical locations.

Population	Chemotype no.	Plant part	Collecting district	Co-ordinates	Altitudes (m-asl)	Dry weight obtained (g)	Yield (%)	Piperine content (mg/g)
Pop 1	PL 1	L	Howrah	22°34′48″N 88°19′46″E	12	0.104	1.04	0.236
	PL 2	S		22°34′48″N 88°19′46″E	12	0.121	1.21	0.272
	PL 3	F		22°34′48″N 88°19′46″E	12	0.229	2.29	4.425
Pop 2	PL 4	L	Kolkata	22°34′03″N 88°22′12″E	9	0.196	1.96	0.316
	PL 5	S		22°34′03″N 88°22′12″E	9	0.234	2.34	0.423
	PL 6	F		22°34′03″N 88°22′12″E	9	0.273	2.73	6.596
Pop 3	PL 7	L	24 PGS (S)	22.1815262°N 88.53780484°E	9	0.189	1.89	3.125
	PL 8	S		22.1815262°N 88.53780484°E	9	0.153	1.53	5.771
	PL 9	F		22.1815262°N 88.53780484°E	9	0.204	2.04	16.362
Pop 4	PL 10	L	24 PGS (N)	22°08′N 88°30′E	15	0.296	2.96	0.423
	PL 11	S		22°08′N 88°30′E	15	0.260	2.6	0.122
	PL 12	F		22°08′N 88°30′E	15	0.323	3.23	2.943
Pop 5	PL 13	L	Nadia	23°24′N 88°30′E	14	0.125	1.25	0.545
	PL 14	S		23°24′N 88°30′E	14	0.082	0.82	4.322
	PL 15	F		23°24′N 88°30′E	14	0.256	2.56	13.302
Pop 6	PL 16	L	Hoogly	22.88°N 87.78°E	10	0.109	1.09	0.109
	PL 17	S		22.88°N 87.78°E	10	0.120	1.2	0.499
	PL 18	F		22.88°N 87.78°E	10	0.128	1.28	3.714
Pop 7	PL 19	L	Bardhaman	23.2324° N, 87.8615° E	30	0.110	1.1	0.136
	PL 20	S		23.2324° N, 87.8615° E	30	0.116	1.16	0.227
	PL 21	F		23.2324° N, 87.8615° E	30	0.226	2.26	11.614
Pop 8	PL 22	L	Malda	25.0108° N, 88.1411° E	17	0.118	1.18	1.242
	PL 23	S		25.0108° N, 88.1411° E	17	0.171	1.71	0.682
	PL 24	F		25.0108° N, 88.1411° E	17	0.171	1.71	3.509
Pop 9	PL 25	L	Darjeeling	26.71°N 88.43°E	114	0.174	1.74	0.472
	PL 26	S		26.71°N 88.43°E	114	0.163	1.63	0.606
	PL 27	F		26.71°N 88.43°E	114	0.169	1.69	6.756
Pop 10	PL 28	L	Jalpaiguri	26.52°N 88.73°E	89	0.230	2.3	2.052
	PL 29	S		26.52°N 88.73°E	89	0.199	1.99	0.493
	PL 30	F		26.52°N 88.73°E	89	0.239	2.39	8.988

*PL Piper longum*, *L* leaf, *S* stem, *F* fruit.

### Method validation

Table 2 provide a summary of all the analytical method validation characteristics for the quantification of bacoside A. The correlation coefficient (r2) was found to have good linearity between 2 and 10 µg/spot. LOD and LOQ were determined to show the method's adequate sensitivity, and they are shown in Table 2.

**Table 2:** Different parameters for HPTLC method validation.

S. no	Parameters	Piperine
1	Linearity range (*µg* /spot; *n* = 12^a^)	2–10
2	Correlation coefficient (*r*^2^)	0.9957
3	Regression equation	Y = 15.476*X + 7911
4	Calculated SD value (CATS software)	2.82
5	^b^ Limit of detection (LOD) (ng) [3 × SD/S]	140
6	^b^ Limit of quantification (LOQ) (ng) [10 × SD/S]	1200
7	*R*_f_ and λmax (nm)	0.63 and 340
Precision and accuracy		
8	Intra-day RSD (%), *n* = 5	0.23
9	Inter-day RSD (%), *n* = 5 (day-1/day-2/day-3)	0.48
Recovery		
10	Amount of standard in fruit samples (µg mg^−1^) containing highest Piperine	13.302
11	Amount of standard added in fruit sample (µg mg^−1^)	17, 34, 51
12	Amount of standard found (µg)	50.58, 67.62, 84.64
13	Recovery (%)	99.98, 100.04, 100.05

^a^Four concentration levels in triplicates.

^b^SD is the standard deviation of the blank response and S is the slope of the calibration curve.

### Precision

Application of 50, 100, and 150% of piperine in plant extract was used to test the developed method's accuracy. To assess the consistency and reproducibility of the findings, the % RSD (coefficient variation) for intra-day and inter-day precision (n = 3 $\times$ 5) was examined. Table 2 displays all results derived from these analytical parameters (average recoveries and intraday and inter-day precision).

### Specificity

Densitometric chromatography and peak purity were used to examine the method's specificity. The correct separation of all the examined substances was demonstrated by densitometric chromatograms. Based on regression values (r2), peak purities of the substances under investigation were decided. Using superimposable spectra, the Rf values of the reference substances were compared to all plant samples and determined to be equal. No impurity or deteriorating products were found during a check of the developed method's specificity.

### Assay for antioxidant activity

#### DPPH assay

**Figure 6:**
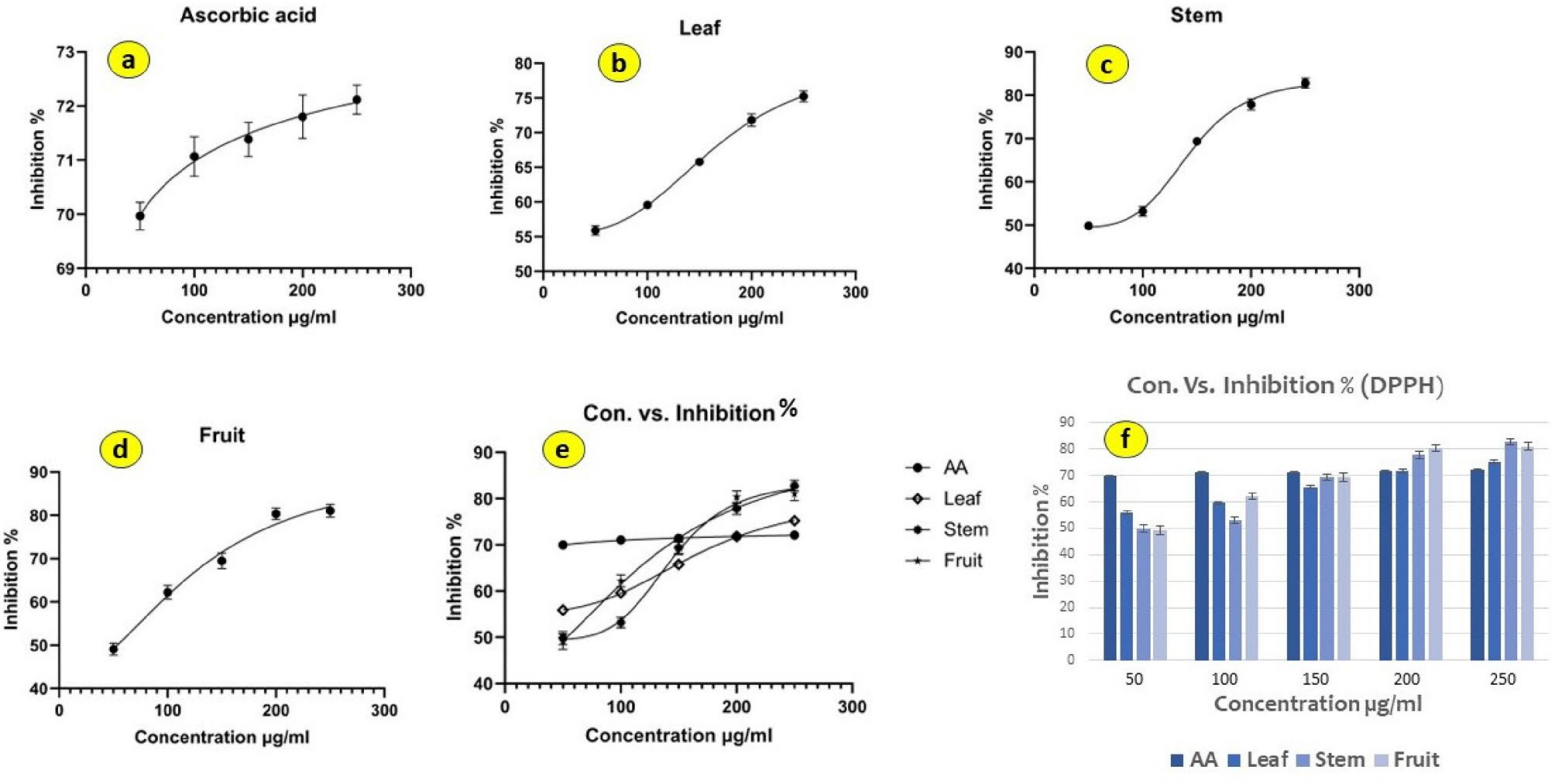
[Inhibitor] vs. response—Variable slopes (four parameters) for DPPH assay: (**a**) Ascorbic acid, (**b**) *P. longum* leaf, (**c**) stem, (**d**) fruit, (**e**) combined slopes, (**f**) bar graph showing % of inhibition of the extracts of different parts of *P. longum*.

The ascorbic acid and tested extracts' inhibition percentages (Fig. 6) and IC_50_ values (the concentration of the sample tested necessary to eliminate 50% of the DPPH radical) are displayed in Table 3. The effectiveness of the *P. longum* plant part extracts may be assessed and compared using these values. In fact, the antioxidant activity increases with decreasing IC_50_ values. *P. longum*'s fruit extract has the highest level of DPPH scavenging ability with an IC_50_ of 124.2 ± 0.24 µg/ml, followed by the stem IC_50_ = 143.3 ± 0.56 µg/ml, then the leaf extract with an IC_50_ of 166.2 ± 0.48 µg/ml. The statistical analysis showed that there is a significant difference between the methanol extracts of *P. longum* and the common antioxidant ascorbic acid. All extracts have low antioxidant capacity compared to the reference standards, according to a comparison between the IC_50_ values of the methanol extracts and the IC_50_ value of the standard antioxidants 2.062 ± 0.13 µg/ml.

**Table 3:** Antioxidant activity of *P. longum* extracts by DPPH and ABTS radical scavenging assay.

Con. (µg/ml)	DPPH assay				ABTS assay			
	AA	Leaf (PL7)	Stem PL8)	Fruit (PL9)	AA	Leaf (PL7)	Stem (PL8)	Fruit (PL9)
	Inhibition %				Inhibition %			
50	69.97 $\pm 0.25$	55.91 $\pm 0.66$	49.85 $\pm 1.39$	49.09 $\pm 1.69$	79.12 $\pm 0.17$	59.93 $\pm 0.45$	56.87 $\pm 0.39$	55.53 $\pm 0.63$
100	71.07 $\pm 0.36$	59.59 $\pm 0.57$	53.19 $\pm 1.13$	62.22 $\pm 1.30$	79.56 $\pm 0.06$	66.88 $\pm 0.50$	67.21 $\pm 0.20$	69.21 $\pm 0.41$
150	71.38 $\pm 0.31$	65.78 $\pm 0.48$	69.36 $\pm$ 1.26	69.48 $\pm 1.60$	79.78 $\pm 0.04$	79.04 $\pm 0.59$	75.98 $\pm 0.38$	79.78 $\pm 0.68$
200	71.80 $\pm 0.40$	71.82 $\pm 0.90$	77.86 $\pm$ 1.29	80.34 $\pm 1.33$	79.98 $\pm 0.02$	81.97 $\pm 0.44$	81.25 $\pm 0.34$	84.56 $\pm 0.61$
250	72.12 $\pm 0.27$	75.23 $\pm 0.78$	82.81 $\pm 1.13$	81.05 $\pm 1.53$	80.11 $\pm 0.03$	84.87 $\pm$ 0.81	83.11 $\pm 0.27$	86.03 $\pm$ 0.49
IC_50_	2.06 $\pm 0.03$	166.2 $\pm 1.12$	143.3 $\pm 1.34$	124.2 $\pm 0.97$	1.98 $\pm 0.54$	119.5 $\pm 0.67$	114.7 $\pm 0.43$	104 $\pm 0.78$

Data are presented as means ± SE (standard error) of three samples of each extract, analyzed individually in triplicate (p < 0.05) (n = 3).

#### ABTS radical scavenging assay

**Figure 7:**
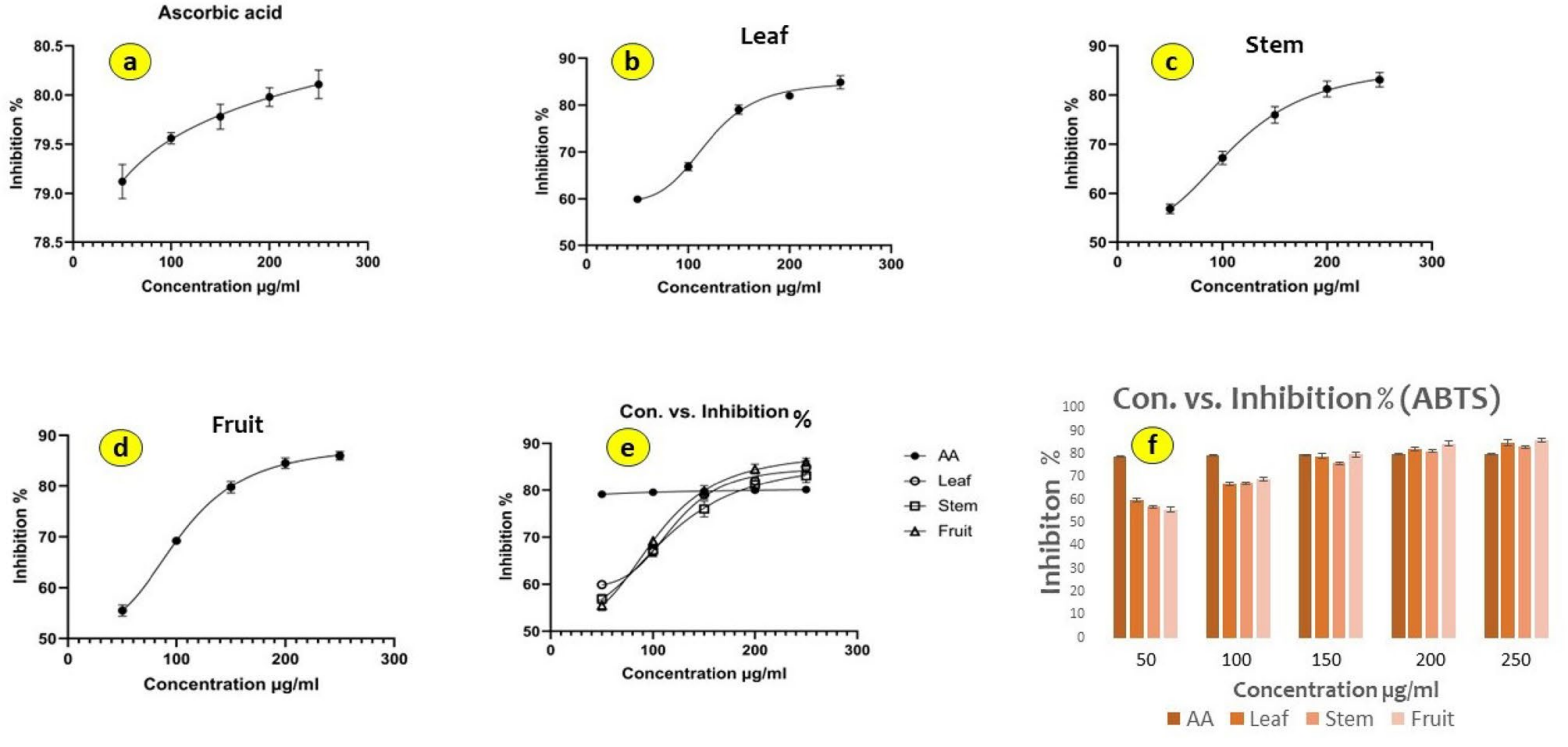
[Inhibitor] vs. response—Variable slopes (four parameters) of ABTS assay (**a**) Ascorbic acid, (**b**) *P. longum* leaf, (**c**) Stem, (**d**) fruit, (**e**) combined slopes, (**f**) bar graph showing % of inhibition of the extracts of different parts of *P. longum*.

In the current work, we assessed the capability of *P. longum* leaf, stem, and fruit methanol extracts (chemotype PL7, PL8 and PL9 of Pop 3) to scavenge ABTS radicals. Comparing the *P. longum* methanol extracts to the ascorbic acid standard, the ABTS radical scavenging activity showed a dose-dependent relationship (Table 3). The scavenging capacity (Fig. 7) of the *P. longum* methanol extracts were 84.87 ± 0.81, 83.11 ± 0.27, and 86.03 ± 0.49% respectively, where AA showed 80.11$\pm 0.03$% inhibition at the highest concentration of 250 µg/ml. The IC_50_ values of the methanolic extracts of PL7, PL8 and PL9 were 119.5 ± 0.67, 114.7 ± 0.43, and 104 ± 0.78 μg/ml a while the standard, ascorbic acid giving a value of 1.98 ± 0.54 μg/ml. The ABTS radical scavenging capacity was found to be in the following order: AA > PL9 > PL8 > PL7.

### Genotoxicity assessment using *Allium cepa* root tip assay

**Figure 8:**
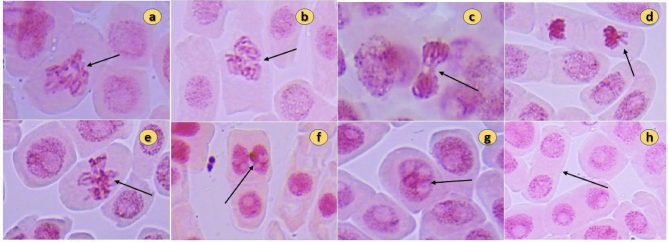
Chromosomal aberrations in A. cepa root tip meristem cells after treatment with *P. longum* extract: (**a**,**b**) multipolarity at metaphase, (**c**) anaphase bridge, (**d**) vagrant chromosome, (**e**) chromosomal stickiness, (**f**) micronucleus, (**g**) multi-nucleolus, (**h**) binucleate cells.

No significant suppression of MI was shown by the *P. longum* (chemotype PL9) plant extract, and no significant reduction in MI was seen with increasing fruit water extract concentration, which was statistically significant in comparison to both controls (positive and negative) (Table 4). A very small number of chromosomal abnormalities (Fig. 8) were present in the plant extract with dose of 20 mg/ml whereas lesser concentrations 10 mg/ml and 5 mg/ml showed very few to no abnormalities. In addition, chromosomal abnormalities such anaphase bridges, multipolarity, and micronucleus formation numbers were also very rare. Though there were few binucleate and multi-nucleolate nucleus were observed. The lowest MI and most frequent chromosomal abnormalities were seen in the sets that had been given the positive control treatment. The Table 5 represents the chromosomal aberrations found in different concentrations of the plant extract.

**Table 4:** Cytological effect and Mitotic index (MI) in *A. cepa* root meristem cells in controls and different concentration of *P. longum* (chemotype PL9) extracts.

Treatment group	Concentration	Total number of cells in each microscopic field	Total number of dividing cells	No of cells in prophase	No. of cells in metaphase	No of cells in anaphase	No. of cells in telophase	Mitotic index $\pm$ SE
Negative control (distilled water)	–	500	38	13	9	10	8	7.6 $\pm 0.14$
Positive control (EMS)	2 × 10^−2^ M	500	12	2	4	3	3	2.4 $\pm 0.09$
PL 9	5 mg/ml	500	35	11	8	9	7	7 $\pm 0.12$
PL 9	10 mg/ml	500	32	9	7	11	5	6.4 $\pm 0.10$
PL 9	20 mg/ml	500	29	6	7	10	6	5.8 $\pm 0.06$

Data are presented as means ± SE (standard error) of five slides of each extract, analyzed individually (p < 0.05) (n = 5).

**Table 5:** Chromosomal aberration in *A. cepa* root meristem tissue after treatment of controls and different concentration of *P. longum* extracts.

Treatment group	Concentration	Multi-polarity	Anaphase bridge	Vagrant chromosome	Stickiness	Micronucleus	Multi-nucleolus	Bi-nucleate cell	% of aberrant cells
	Chromosome aberration per 500 cells								
Negative Control (Distilled Water)	–	–	–	–	–	–	–	–	–
Positive Control (EMS)	2 × 10^−2^ M	0.8 $\pm 0.$ 24	1.4 $\pm 0.2$	–	$0.6\pm 0$.14	0.4 $\pm 0.26$	0.8 $\pm 0.19$	1 $\pm 0.21$	5.5 $\pm 0.11$
PL 9	5 mg/ml	0.2 $\pm 0.31$	0.2 $\pm 0.26$	–	0.2 $\pm 0.33$	–	–	–	0.6 $\pm 0.09$
PL 9	10 mg/ml	0.4 $\pm$ 27	0.6 $\pm 0.42$	–	0.2 $\pm 0.53$	–	–	–	1.36 $\pm 0.07$
PL 9	20 mg/ml	0.6 $\pm$ 0.44	1 $\pm 0.31$	0.4 $\pm 0.51$	0.2 $\pm 0.19$	0.4 $\pm 0.28$	0.4 $\pm 0.41$	1.2 $\pm 0.58$	3.46 $\pm 0.12$

Data are presented as means ± SE (standard error) of five slides of each extract, analyzed individually (p < 0.05) (n = 5).

## Discussion

The elite germplasms of any plant species must be identified through chemotaxonomic research in order to pursue the commercial development of a certain metabolite. The current densitometric HPTLC technique shows substantial variation of the marker chemical piperine among 30 chemotypes of *P. longum* from 10 distinct populations. This information may be useful for choosing high yielding elite *P. longum* germplasms over poor yielding ones for industrial-scale commercial use. Temperature, altitude, soil, UV radiation, etc. are just a few examples of the many variables that affect the quality and quantity of secondary metabolite content in botanicals^17^. *P. longum* chemotypes were collected for this study from 10 different districts in West Bengal, India, separated geographically. As a result, the distinct geographical and climatic zones of *P. longum* populations' natural environment may clearly be linked to the intra-specific quantitative variation in the biomarker piperine in those populations. In a number of chemotaxonomic investigations conducted in plants previously, the secondary metabolites stevioside and rebaudioside A in *Stevia rebaudiana*, for example, showed chemotype dependent changes^18^, aristolochic acid in *Aristolochia indica*^19^, secoiridoid glycoside like mangiferin and secoiridoid glycoside like swertiamarin in *Swertia* species^20^, diterpene, triterpene, glucoside sterols from *Clerodendrum* sp.^21^ and others. Additionally, it was discovered that the content of secondary metabolites varied depending on eco-geographical region^22^, latitude^23^, altitudinal^24^, exposure to UV-radiation^25^, temperature^26^, duration of sunshine^27^ etc. Tissue specific variation in primary and secondary metabolites was also reported^28^. As a result of their exposure to and interaction with a number of environmental and ecological factors, plant genotypes are known as chemotypes because they exhibit qualitative and quantitative alterations in biomarker molecules^17^. Piperine was previously quantified in *P. longum* fruits only using HPTLC^8,9^. Recent studies showed that Piperine was quantified in *P. longum* and *P. nigrum* extracts using Soxhlet and Super critical fluid extraction methods^9^, accelerate solvent extraction method^29^, quantified piperine from ayurvedic formulations and compared with the piperine content of root (0.29 ± 0.42 mg/g) and fruit (1.48 ± 0.95 mg/g) of *P. longum*^10^. Different commercial brand of *P. nigrum* seed were analysed by normal and reversed phase HPTLC to quantify the piperine content by using two different extraction methods: traditional and ultrasound^30^. Gaur et al., also quantified six alkamides including piperine from *P. longum* using various separation solvent system^31^, but there is no report of geographical and tissue specific variation of piperine content. Our study investigates and quantifies piperine content from different parts of *P. longum* from different geographical locations. This study helps in identifying the highest piperine yielding chemotype so that it can be further grown commercially to use in industrial purpose. We found piperine content in fruit chemotype PL9 as higher as 16.362 mg/g which very high amount according to the previous studies. Our study also investigates its anti-oxidant activity as well as toxicological activity which will help to understand that this plant has bioactivities and it is safe to use.

In this study the biomarker piperine is found to be present in all chemotypes and the content of piperine varies in all the collected regions. The highest amount of piperine found in South 24 Parganas which is near the Ganges River, while least amount of piperine found from Kolkata which is an urban area. Different levels of alkaloid piperine were present in plant samples collected from similar geographic areas. Tissue specific variation of piperin content also showed significant result. The fruit chemotypes contain high amount of piperine (16.36 mg/g) following the stem and leaf. Therefore, choosing high yielding elite chemotypes can remove the variability in the quality of the secondary metabolites that are harvested from medicinal plants and allow them for commercial cultivation and production of products that are commercially viable. Piperine, a key alkaloid biomarker of *P. longum*, was measured in the current work using a proven HPTLC method to identify the plant's elite chemotypes, which may then be further exploited or domesticated for commercial use.

By serving as natural antioxidants, plants with beneficial phytochemicals may complement what the body requires^32^. According to numerous research many plants are a great source of antioxidants such as of antioxidants include the vitamins E, A, and C, as well as phenolics, flavonoids, lignins, and tannins that are present in plants^33^. The presence of various phytochemicals contributes to the effectiveness of conventional formulations by acting as powerful antioxidants in plant extracts or herbal preparations^34^. Numerous chronic inflammatory diseases, neurological diseases, metabolic disorders, cancer, and cardiovascular disease are thought to have oxidative stress as a primary or secondary cause^35^. The findings of the DPPH and ABTS tests (% inhibition) performed in the current investigation showed that plant extracts had promising antioxidant potential. The plant extracts shown moderate antioxidant activity in the DPPH and ABTS free radical scavenging tests. This activity could even be seen visually by watching the gradient-wise production of light blue colour in the DPPH and ABTS assays. The piperine contents of *P. longum* (PL 9) were found highest in the fruit, then stem and the lowest in the leaf. The IC_50_ values for DPPH and ABTS assay were lowest in the fruit, then stem and higher in the leaf. The more effective a compound is at scavenging DPPH or ABTS, suggests a higher level of antioxidant activity, indicated by a lower IC_50_ value^36^. Antioxidant activity is characterised as being extremely potent when the IC_50_ is less than 50 ppm, strong when the IC_50_ is between 50 and 100 ppm, moderate when it is between 101 and 250 ppm, weak when it is between 250 and 500 ppm, and classed as inactive when it is greater than 500 ppm^37^. 1 ppm corresponds to 1 µg/ml. By blocking or quenching free radicals, reactive oxygen species, and hydroxyl radicals, piperine, which has an anti-inflammatory action, has been shown in vitro trials to protect against oxidative damage^38^. Thus, it can be concluded that plant part containing higher amount of piperine exhibits more antioxidant activity.

The test method for chromosomal alterations in *A. cepa* is often used in the literature as a bioindicator for the assessment of cytotoxicity, chromosomal size, fast cell division, and a small number of chromosomes provide for a better understanding of structural and numerical alterations as well as the genotoxicity and protective effects of chemical substances^39,40^. This test has been used for a long time to examine the mutagenic effects of poisons and other harmful substances, including plants that can cause cytotoxicity and genotoxicity^41,42^. The *A. cepa* root tip test was used in the current work to conduct genotoxic investigations on the *P. longum* aqueous plant extract (PL9 chemotype). In this study we did not observe significant cytotoxicity or genotoxicity as well as reduction in mitotic index of the tested extracts. Though the highest concentration used 20 mg/ml showed some chromosomal aberrations which is not noteworthy in comparison to the positive control EMS (Ethyl methanesulfonate) may be because piperine and the other compounds present in *P. longum* did not affect the mitosis stage and chromosomes of *A. cepa* root tip cells. The monofunctional ethylating agent EMS was utilised as the positive control in this work and has been shown to have mutagenic effects in a variety of genetic experiments on viruses, animals, and plants^43^. Piperine was tested for its genotoxicity in vivo and also by in vitro MNT and Ames test, where piperine did not increase the micronucleus frequency and proved that it was not at all genotoxic^44^. Yadav et al. in their investigation found that *P. longum* showed genoprotective activity through the reduction of oxidative stress, DNA damage and doble strand break, neurotoxicity as well as hepatotoxicity in cyclophosphamide induced genotoxicity^45^. This studies prodive evidence for genoprotective activity of *P. longum* rather than for genotoxic activity. *P. longum* is popularly consumed as a culinary spice and medicinal plant as well as it has ethno-veterinary use throughout Indian subcontinent, Middle Eastern countries, Sri Lanka, and the Americas^38^. This Ayurvedic herb Pippali (*P. longum*) is popularly consumed orally as treatment of cough, bronchitis, cold, as antidote in scorpion sting and snake biting, and as contraceptive tonic and other formulations^1,46,47^. The findings supported the extensive use of the plant as a culinary spice and in folk remedies throughout the globe since they showed that no significant mitotic and chromosomal abnormalities occurred even at greater doses of the applied plant extracts.

For the purpose of identifying elite chemotypes, a technique was established in the current study that was optimised and validated for HPTLC-quantitation of the bioactive compound piperine in natural populations of *P. longum* in India (West Bengal). The innovative, straightforward, accurate, and exact HPTLC technique was successfully established for the detection and quantification of piperine in 30 chemotypes from 10 populations with 3 tissue-specific variants (Leaf, stem, and Fruit). The study demonstrated intra specific variations in piperine content in the collected chemotypes, which varies according to the geographical regions, where the fruit chemotypes found to contain higher amount of piperine. The study further analysed the antioxidant property of the plant part, and the free radical inhibitory activity in DPPH and ABTS assay of the chemotypes positively correlates with the piperine content. The *A. cepa* root tip assay, also validated the plant’s lack of genotoxic property, and demonstrated that it is widely accepted as a popular medicinal and culinary spice among ethnic as well as modern populations pantropically. This is the first report on HPTLC method validation and quantification of piperine from *P. longum* with geographical as well as tissue specific variation. The phytochemical profile that the current study provides from the collected *P. longum* plant accessions, would be advantageous to the pharmaceutical industry for mass propagation and commercialization. Its pharmacological effectiveness was further supported by its’ promising antioxidant activity, and a genotoxicity study demonstrated its safety for consumption and for use as medication.

## Materials and methods

### Plant materials

Different plant parts (leaf, stem, fruit) of *P. longum* were collected from 10 different regions of West Bengal, India (Fig. S2) including both local cultivated and medicinal plant garden. Fresh plant parts were collected and bought to the laboratory. Dr. Avinash Mundhra, Assistant Professor, Department of Botany, Rishi Bankim Chandra College, West Bengal, India, identified the plant specimens. For reference in the future, a voucher specimen (PL01) was stored at the departmental herbarium in the Department of Life Sciences at Presidency University.

### Ethical approval

Authors confirm that the use of plants in the present study complies with international, national and/or institutional guidelines. We gained appropriate permissions for collection of plant specimens from respective garden authorities and/or owners.

### Chemicals and reagents

Piperine analytical standard, DPPH (1,1 diphenyl-2-picrylhydrazyl), ABTS (2,2'-Azino-bis (3-ethylbenzothiazoline-6-sulfonic acid) diammonium salt, MTT [3-(4,5-dimethyl-thiazol-2-yl)-2,5-diphenyl-tetrazolium bromide] were purchased from Sigma-Aldrich, India. Ascorbic acid, potassium persulphate was bought from Himedia laboratories.

### Plant extract and standard solution preparation

All the plant parts were shed dried and powdered used grinder separately, passed through a 80-mesh sieve. For extraction 1-g dried powder of each sample were weighed accurately and macerated with methanol for 24 h in a shaker (Lab X incubator shaker) in room temperature. The extracts were filtered via Whatman filter paper (grade-1, 90 mm), and a rotary evaporator was used to evaporate the solvent. Each final extract was weighed, redissolved in methanol (10 mg/mL), and kept in a refrigerator at 4 C for further HPTLC analysis. A precise 5 mg of reference standard piperine (Sigma Aldrich, India) was weighed out and dissolved in 5 mL of methanol to create the standard piperine solution.

### Chromatographic condition

Chromatography was carried out on HPTLC plates of 10 cm × 20 cm that were precoated with 0.25 mm layers of silica gel 60 F254 (Merck, Germany). The HPTLC system CAMAG (Muttenz, Switzerland) was composed of a CAMAG TLC scanner-3 with software (Win CATS 3) and an Linomat-V automatic sample applicator. For calibration curve, piperine standard solution (2, 4, 6, 8, 10 µl) were applied to the plates in 6 mm wide bands using the automatic sample applicator Linomat-V ["Linomat5200109" S/N 200,109 (1.00.13)] (with nitrogen flow) that was supplied with a Hamilton syringe (100 µL) with a delivery rate set at 150 nl/s. The plant samples were also applied to the plates in 6 mm wide bands of 10 ml at a delivery rate of 150 nl/s. The plates were developed at a distance of 8.0 cm in in linear ascending condition in a CAMAG twin-trough glass chamber (20 × 10 × 4 cm) chamber pre-saturated with mobile phase vapour using toluene:ethyl acetate:diethyl ether (6:3:1 v/v/v) as the mobile phase at room temperature 22 ˚C (± 2 °C) and relative humidity of 50%. Plates were dried using a hot air drier after development up to 75 mm, and then clear bands were seen under UV (UV cabinet with dual wavelength UV lamp) at λ = 254 nm. The plates were scanned at 254 nm (the maximum wavelength for piperine) using WinCats 3 software ["Scanner_200208" S/N 200208 (2.01.02)] and a CAMAG TLC scanner 3. A graph was created by plotting the average peak area versus the amount of piperine after this procedure was repeated five times. The parameters for densitometric scanning were adjusted to a 4.00 × 0.30 mm slit size, 20 mm/s scanning speed, and 100 µm/step data resolution.

### Preparation of standard curve

Using peak areas of the standard piperine (2, 4, 6, 8, 10 µl) versus its’ pertinent concentrations, a calibration curve was prepared (Fig. S1). In all of the plant samples that were put to the test, the yield of the reference compound was calculated using a regression equation and the associated peak area.

#### Validation

In accordance with ICH (International Conference on Harmonization council) (ICH, 2005) recommendations, the method was validated by evaluating the peak purity, precision, specificity limit of detection (LOD), limit of quantitation (LOQ), instrument precision, repeatability, reproducibility and piperine’s percentage recovery from samples.

##### Precision

To test the instrument's precision, the same spot for piperine (100 ng/spot) with n = 5 was employed. Three different concentrations of the reference substances were tested for repeatability on the same day (intra-day) and reproducibility (inter-day) three consecutive days, respectively. The findings of these seven different analyses are represented as mean % RSD (Relative Standard Deviation).

##### Limit of detection and limit of quantification

LOD and LOQ values were assessed for the signal-to-noise (S/N) ratio using various concentrations of reference substances and methanol as a blank. The difference between LOD and LOQ was measured as 3,1 (SD/S) and 10,1 (SD/S), respectively. Here, S denotes slope and SD represents standard deviation of the Y-intercept from the regression line.

##### Specificity

For more specificity, the peak purity of the standard compound and the plant samples were matched at the peak's beginning, at the peak maxima, and at the peak end. In addition, marker compound and the overlay spectra of the isolated bands of the plant samples were compared. The accompanying Rf values were verified using the separated band of the standard chemical with plant samples in their scanning densitometric chromatograms.

### Antioxidant activity

#### DPPH radical scavenging assay

To assess the antioxidant capacity of the *P. longum* plant extracts a modified DPPH assay, as published by Chavan et al. 2014^48^, was used. DPPH free radical solution was obtained by mixing 0.1 mM DPPH with methanol using a magnetic stirrer for 12 h at 25 °C. An extract dilution series with the concentration range 250–50 µg/mL was prepared using extracts of Pop3 (Chemotypes PL7, PL8, and PL9) as this population contains highest amount of Piperine. The DPPH radical solution (1 mL) was added to 3 mL of the extracts and standard solution (50, 100, 150, 200 and 250 µg/ml), separately and left to incubation in dark for 30 min at 25 °C. The absorbance was measured at 517 nm. Decreasing absorbance demonstrates DPPH free radical scavenging capacity.

The control consists of 3 ml of methanol and 1 ml of DPPH solution. The positive control is represented by a solution of standard antioxidants, ascorbic acid, whose absorbance is measured under the same conditions as the sample tested.

The percentage inhibition of DPPH is calculated according to the following formula:

Percentage of radical scavenging activity = $\frac{Abs\, of\, control-Abs\, of\, sample}{Abs\, of\, control}\times 100$ where, Abs = Absorbance (Brand-Williams et al., 1995)^49^.

#### ABTS assay

Using the ABTS cation decolorization assay, the ABTS radical scavenging capacity was assessed according to the method described by Tuyen et al., 2017^50^ with some modifications 0.7 mM ABTS and 2.45 mM K_2_S_2_O_8_ (Potassium persulfate) solution were reacted for 12 h in dark to procure experimental solution of ABTS cation radical. With the help of 0.1 M phosphate buffer (pH 7.4) dilation, the absorbance of the control solution was adjusted to 0.7 ± 0.1 at 734 nm. An extract dilution series with the concentration range 250–50 µg/mL was prepared using extracts of Pop3 (Chemotypes PL7, PL8, and PL9) as this population contains highest amount of Piperine. 30 µl of each sample and standard ascorbic acid at different concentrations (50 to 250 μg/ml) was added to 1 ml of the ABTS solution and mixed vigorously. The sample volume was increased to 4 mL by adding buffer solution. The absorbance at 734 nm was measured after the reaction mixture was allowed to stand at room temperature for 6 min. The reduction in sample absorption was used to calculate the ABTS· + radical scavenging activity. The ABTS scavenging effect was calculated as per the equation:

Percentage of ABTS scavenging effect =$\frac{Abs\, of \,control-Abs\, of\, sample}{Abs \,of\, control}\times 100$ where, Abs = absorbance.

### Genotoxicity assessment

#### Preparation of the aqueous extracts of *P. longum* plants

To prepare a stock solution (10%), 20 gm of the powdered sample was added to 200 mL of distilled water, stirred thoroughly, and then heated for 10 min in a beaker with a lid. After then, the solution was cooled down at room temperature. The extract was filtered using Whatman No. 1 filter paper. Workable solutions (5, 10 and 20 mg/mL) were created by gradually diluting the stock. At the outset of each trial, all of the extracts were freshly produced.

#### *Allium cepa* root tip assay

To investigate the genotoxicity of the high piperine-yielding *P. longum* population chemotype (PL9), the methodology described by as As¸kinÇelik and Aslantürk, 2010^51^ was used, with a few minor modifications. Distilled water was used as a negative control, and ethyl methane sulfonate (EMS, 2 × 10^–2^ M) was used as a positive control. After 6 h incubation in the controls and the water extract (5, 10 and 20 mg/mL) of *P. longum* plants, the root tips of *A. cepa* were cut off from the onion bulbs and fixed at 4 °C in a solution of ethanol: glacial acetic acid (1:3 v/v) for 16 h. The roots were then placed in a solution of 70% (v/v) ethanol and kept in the refrigerator for further analysis. Following the hydrolysis of the root tips using 1 N HCl for 2 min, and staining the root tips in aceto-orcein [2% (w/v)], slides were prepared using the squashing technique. Each slide was observed under 40 × 10 magnification of a compound microscope (Olympus) with (n = 5 in each set). Mitotic-index (MI), which is the percentage-based comparison of the total number of dividing cells to the total number of cells in a given microscopic field, was used to measure genotoxicity. Different chromosomal aberrations such as anaphase bridges, multipolarity, binucleate cell, multi-nucleolar nucleus, micronucleus etc. were counted and the MI for the same are mentioned in Tables 4 and 5.

### Statistical analysis

All the results procured from the HPTLC screening of the phytochemical and antioxidant assays were run three times with 19 samples in triplicate for each experimental set. Fisher's least significant difference test was used to determine the significance of differences between means. Mean values are denoted as mean SE. Three times, calculations were done using mean values and standard error. The data is shown as mean $\pm$ SE. Student's t-test usage offered statistical significance. P < 0.05 values were regarded as significant. Analysis of variance (ANOVA) was carried out for antioxidant assay as well as *Allium cepa* root tip assay using SPSS software. The tables were made with Microsoft Excel and graphs were made by Graphpad Prism software.

### Supplementary Information

Supplementary Information.

## Data Availability

Authors provided all chromatograms in the manuscript and supplementary file. If anything else required, the corresponding author will provide the datasets used and analysed during the current work upon reasonable request.
