# Supplementary material for: Tissue-specific variations of piperine in ten populations of Piper longum L.: bioactivities and toxicological profile
Source: Sci Rep. 2024 Mar 1;14:5062. doi: 10.1038/s41598-024-52297-9 (PMC10904381; doi:10.1038/s41598-024-52297-9)
Supplement: Supplementary file 1 — Supplementary Information. [file 41598_2024_52297_MOESM1_ESM.docx]

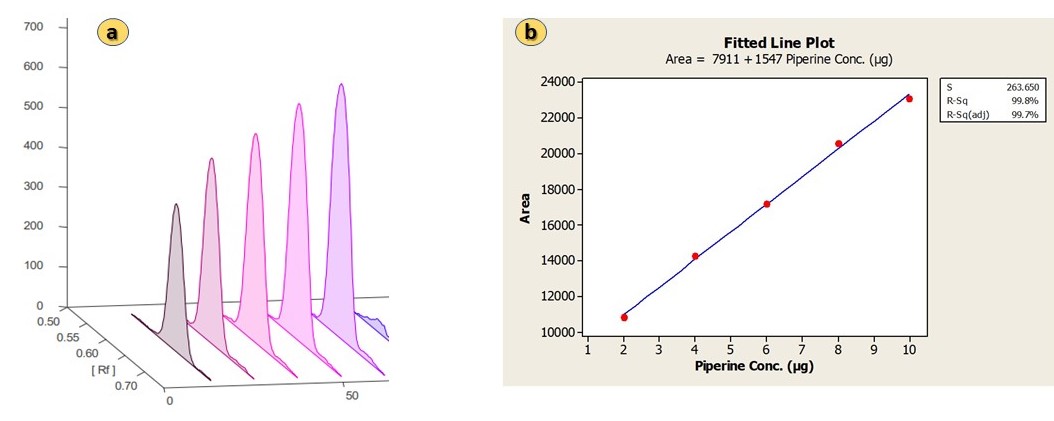


**Fig. S1.** HPTLC chromatography a. 3D densitogram of standard piperine, b. calibration curve

**Figure S2.** Geographical location of collected *P. longum* chemotypes


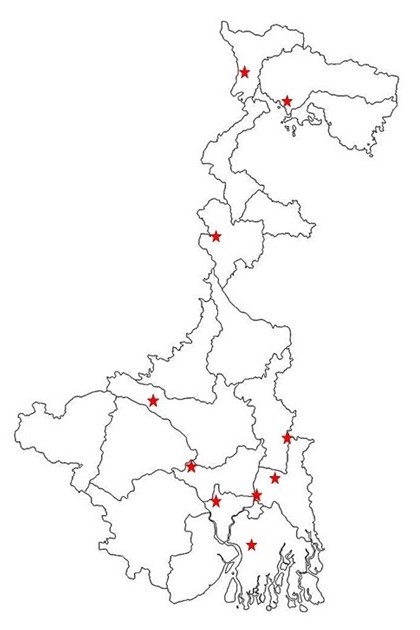


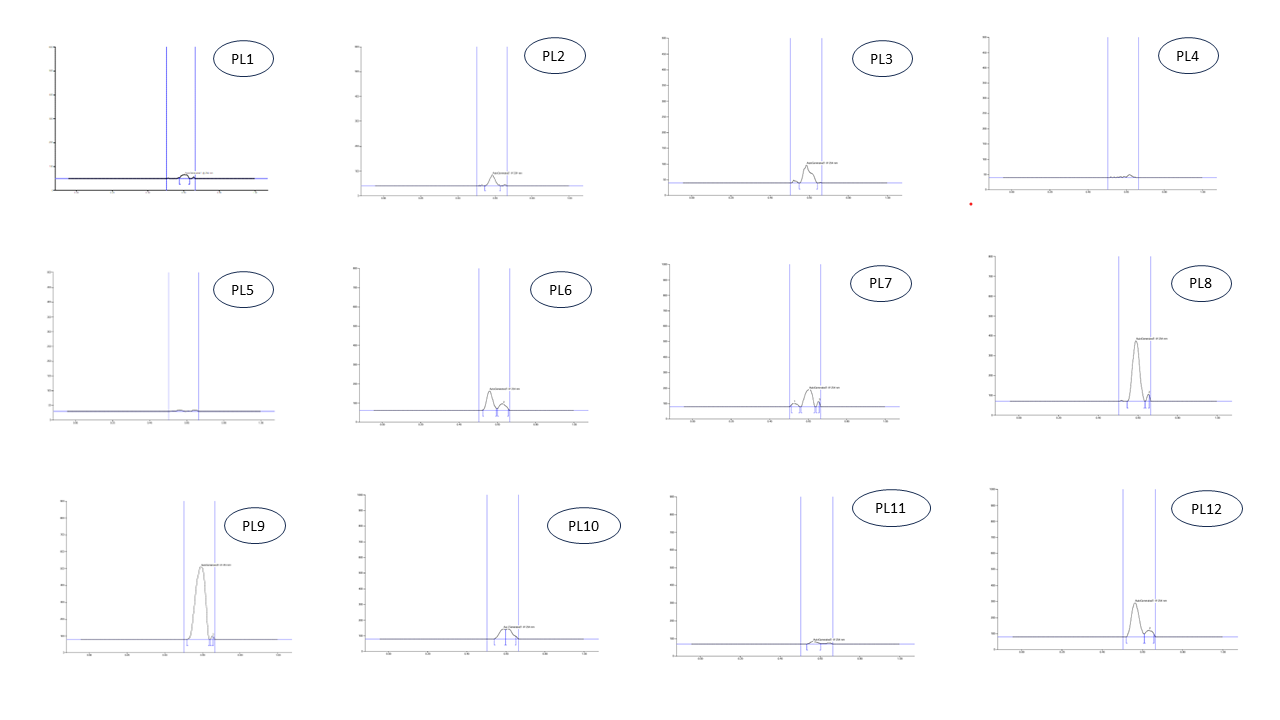


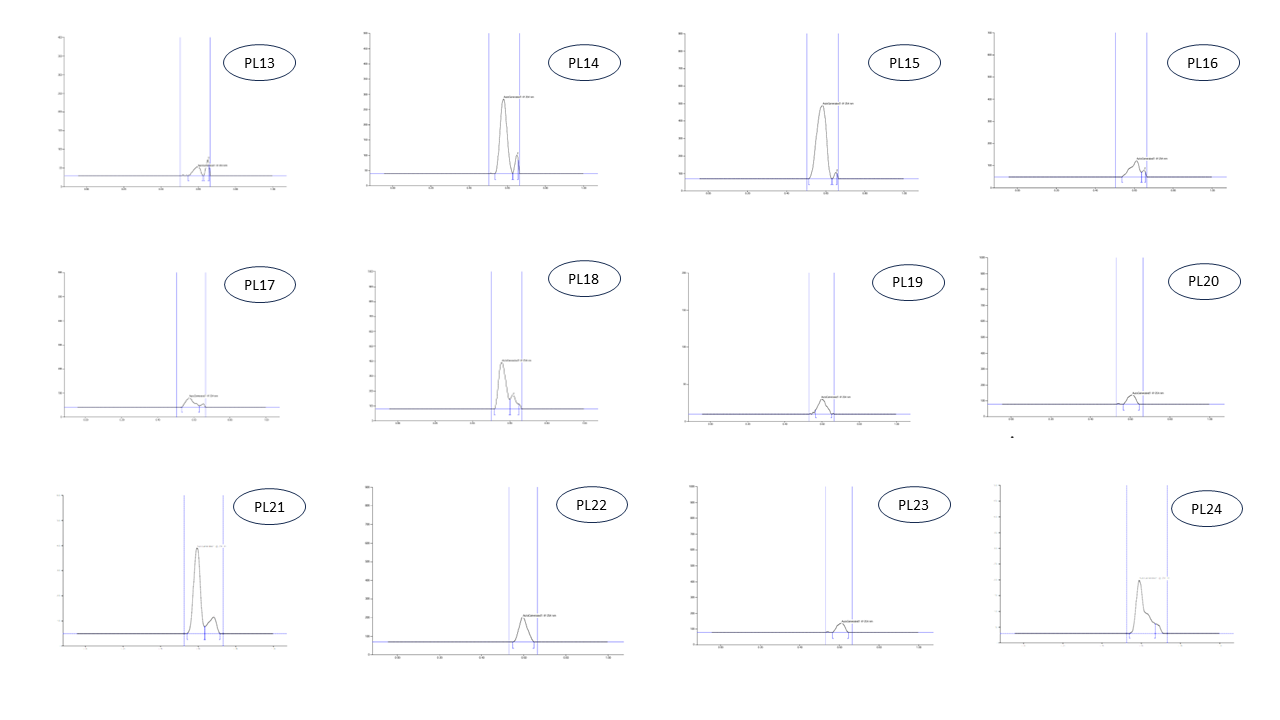


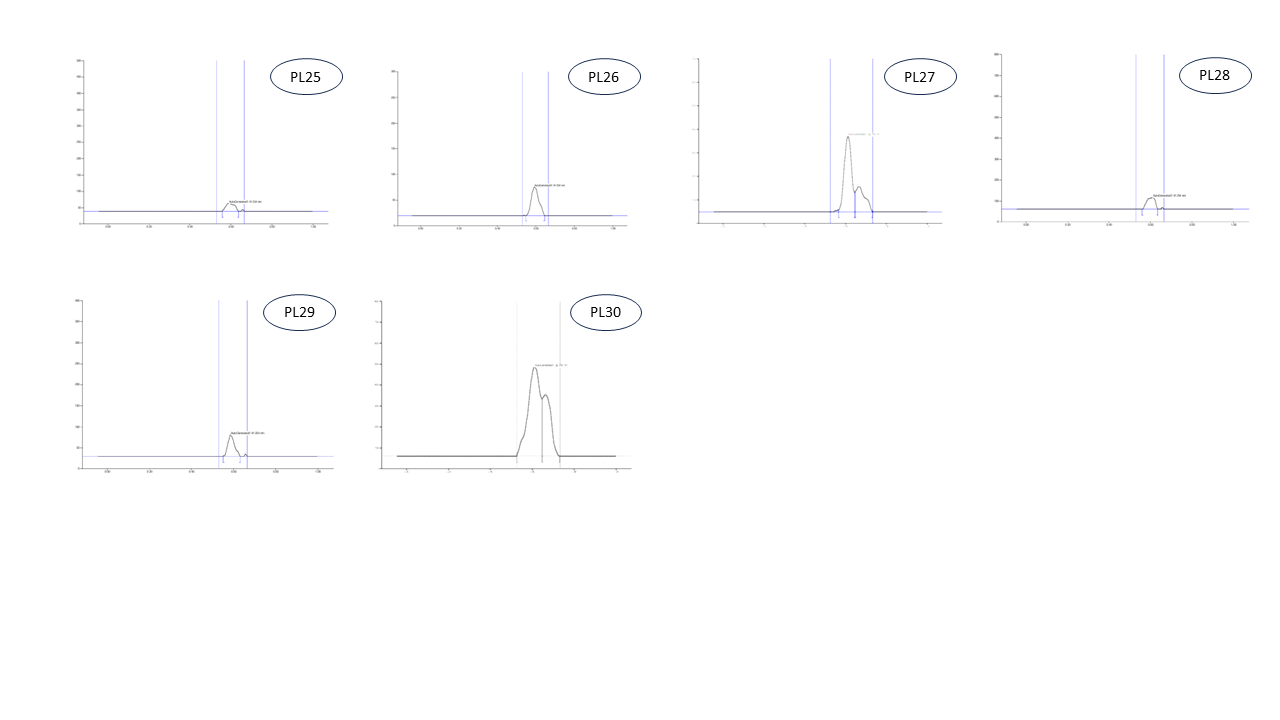


**Figure S3.** Chromatograms of all the chemotypes of *P. longum*
